# A Compact Convolutional Neural Network for Surface Defect Inspection

**DOI:** 10.3390/s20071974

**Published:** 2020-04-01

**Authors:** Yibin Huang, Congying Qiu, Xiaonan Wang, Shijun Wang, Kui Yuan

**Affiliations:** 1 Institute of Automation, Chinese Academy of Sciences, University of Chinese Academy of Sciences, Beijing 100190, China; huangyibin2014@ia.ac.cn (Y.H.); wangsj_ucas2012@163.com (S.W.); kui.yuan@ia.ac.cn (K.Y.); 2 Civil Engineering & Engineering Mechanics Department, Columbia University, New York, NY 10024, USA; cq2192@columbia.edu

**Keywords:** surface defect inspection, convolutional neural network, machine vision

## Abstract

The advent of convolutional neural networks (CNNs) has accelerated the progress of computer vision from many aspects. However, the majority of the existing CNNs heavily rely on expensive GPUs (graphics processing units). to support large computations. Therefore, CNNs have not been widely used to inspect surface defects in the manufacturing field yet. In this paper, we develop a compact CNN-based model that not only achieves high performance on tiny defect inspection but can be run on low-frequency CPUs (central processing units). Our model consists of a light-weight (LW) bottleneck and a decoder. By a pyramid of lightweight kernels, the LW bottleneck provides rich features with less computational cost. The decoder is also built in a lightweight way, which consists of an atrous spatial pyramid pooling (ASPP) and depthwise separable convolution layers. These lightweight designs reduce the redundant weights and computation greatly. We train our models on groups of surface datasets. The model can successfully classify/segment surface defects with an Intel i3-4010U CPU within 30 ms. Our model obtains similar accuracy with MobileNetV2 while only has less than its 1/3 FLOPs (floating-point operations per second) and 1/8 weights. Our experiments indicate CNNs can be compact and hardware-friendly for future applications in the automated surface inspection (ASI).

## 1. Introduction

For a long time, product quality control relies on manual examination in the manufacturing field. In the past several decades, automated surface inspection (ASI) have become more widespread as hardware and software technologies have developed rapidly. To lower labor costs and enhance examination efficiency, more factories start to employ embedded machines for product inspection. ASI platforms use special light sources and industrial cameras to capture the images of the product surface, and machine/computer vision technologies to filter out defective products, which can reduce labor greatly. High-performance cross-product ASI algorithms are urgently needed in manufacturing.

Common surfaces in the manufacturing industry include steel [1,2], glass [3], fabric [4], wood board [5,6], and so forth. Most proposed defect inspection algorithms for these surfaces are traditional classification or segmentation methods. For traditional classification methods, for example, Gabor [7], LBP (local binary pattern) [8], GLCM (gray-level co-occurrence matrix) [9] and HOG (histogram of oriented gridients) [10] features of the defects are extracted, then the defects are classified by machine learning classifiers, such as SVM (support vector machines) [1], Random Forests [11], AdaBoost [10], and so forth. Traditional segmentation methods would partition pixels into regions, with the intention to emphasize the regions corresponding to surface defects. For example, Reference [6] firstly preprocesses images using convex optimization, then segments wood defects with Otsu segmentation method. Reference [12] segments fabric defects by the Sobel and the watershed algorithm.

These traditional methods involve image preprocessing, feature extraction, feature reduction, and classifier selection, which needs the experience of experts. Besides, most surface defects are tiny and very similar to their background, even the inspection for a single type of defect can be very difficult for these traditional methods. Although traditional algorithms are designed for specific surfaces, effective measures such as recall, precision, and accuracy can barely achieve the standard of industrial applications. This long-existing bottleneck has been restraining the growth of ASI.

Convolutional neural networks (CNNs) greatly stimulate the development of ASI [3,13,14,15] as deep learning methods in computer vision are achieving the state-of-the-art in recent years. As end-to-end solutions, CNNs also simplify the procedures of ASI. For example, a fully convolutional network (FCN) can segment surface defects by supervised learning [16], without any extra pre-process or post-process.

A notorious limitation of CNNs is computation. In many cases, expensive GPUs with high computing capabilities are necessary to support large workloads. However, in considering manufacturing cost, most core computing resources of ASI platforms are mainly the low-power CPUs of industrial personal computers (IPC), or even FPGAs (field programmable gate array). To take advantage of CNNs in ASI while continuing to use these low computing capability processor units, converting into compact CNNs is a workable option. Meanwhile, a CNN with high performance needs large datasets to train, while there exist few online datasets for surface defect images.

To address these limitations, this work introduces our novel lightweight CNN, the compact design remarkably reduces the number of weights and computation while still achieving a high performance. We also investigate the availability of a generic solution for ASI. That is, we apply our compact model to multiple types of surfaces. Following proper training strategies, our model has satisfactory results for different materials with small training sets. It is worth pointing out that the defect pixels only take a small proportion of the whole image, therefore, the backpropagation gradient of defects will be overwhelmed by the background gradient, and subsequently cause low accuracy. Moreover, the images with defects are usually far less than the non-defect, the networks do not have sufficient data to learn the defect features, resulting in accuracy significantly decreases. To tackle this problem, we apply the gradual training strategy where the defective regions are intentionally enlarged with specific scales. Our main contributions are:

- Propose an application-oriented deep neural network for cross-product ASI. Our model obtains the state-of-the-art in both classification and segmentation.
- Modify the residual bottleneck with small kernels and depthwise convolution, build a lightweight backbone and decoder. Our model significantly reduces the number of weights, computation and time cost.
- Obtain ∼5% true positive rate improvement on tiny defect detection, by training our model with gradual training and a fine-tuned strategy.

## 2. Related Work

### 2.1. Compact Design

Intuitively, increasing parameters, computation or the convolution layer may improve the accuracy of CNN models. Table 1 reveals the performance result of the well-known models trained on ImageNet (ILSVRC-2012-CLS) [17]. In the table, *W* is the number of weights (M, or $\times 10^{6}$); *F* is the FLOPs ($\times 10^{6}$) evaluated with TensorFlow [18], which accumulates the computation including convolution, pooling, regularization, batch normalization, and all other operations defined in the graph. The statistics seemingly indicate that a model with more FLOPs and weights is more likely to obtain higher accuracy. However, we observe some cases against this assumption—(1) Inception-V4 [19] still slightly outperforms ResNetV2-200 although it only has the 1/2 FLOPs and the 2/3 weights of ResNetV2-200 [20]. (2) MobileNetV2@1.4 has much fewer weights than VGG-16 while it achieves a higher accuracy by 4%.

Interestingly, many compact deep neural network (DNN) models can still achieve an excellent performance on image classification, such as SquezeNet [21], Compact DNN [22], MobileNetV1 [23], ShuffleNet [24], and MobileNetV2 [25]. Some popular applications of object recognition include gesture recognition [26], handwritten Chinese text recognition [27] and traffic sign classification [28]. Now, the demand for compact models on these applications prompts an increase. Compact models use specific convolution methods to reduce redundant weights, for example, 1×1 convolution, group convolution, and depthwise separable convolution. Compact models are also used in surface defect inspection, for instance, Reference [29] introduces a compact CNN to perform defect segmentation and classification simultaneously.

### 2.2. Transfer Learning

Transferring the knowledge from a large scale source dataset to a small one has proved to be a meaningful practice. Reference [30] tried to investigate the best approach for transferring knowledge. In their experiment, 13 ImageNet classifiers were trained via three different methods—(1) random initialization; (2) fine-tuned from ImageNet-pretrained weights; (3) freeze CNN as fixed feature extractors. Then these classifiers were assigned to 12 small datasets. The study shows that fine-tuned models significantly outperform others. At the same time, those models that achieve higher accuracy on ImageNet also exceed others on different datasets. A considerable large source dataset is a key factor of using transfer learning to improve the target dataset. In DeeplabV3+ [31], the source dataset for model pre-training is JTF-300M [32], which is 300 times larger than ImageNet. Using this super large dataset, researchers pretrain a 65-layer Xception [33] model, then fine-tune a segmentation model based on PASCAL VOC 2012. The result suggests these strategies have remarkably leveraged performance, their model won the best-recorded mIOU (Mean Intersection over Union). In the practice of surface inspection [15], a fine-tuned ImageNet pretrained VGG-16 is used as a patch classifier on surface defect inspection. The experiment shows that fine-tuning all layers is the best valid way for models to achieve high accuracy. Training neural networks with the fine-tune strategy can gain much better results than with random initialization. Based on these studies, we opt to utilize the fine-tune strategy.

### 2.3. Model Compression

Deep neural networks are computationally expensive and memory expensive, while hardware resources are very limited in reality. For example, in mobile applications, the application size and graphics computing resources are constrained. To address this issue, model compression has been introduced to help models easier to deploy embedded systems by reducing redundant parameters and computation [34]. In early work, pruning [35] reduces the number of redundant connections; quantization [36,37] compresses the floating-point operations by quantifying 32-bit floating-point parameters to 16-bit, 8-bit or even 1 bit fixed-point; Low-rank decomposition [38] removes the redundancy in the convolution kernels through computing the low-rank tensor decomposition; Knowledge distillation [39] trains small student network with the knowledge of a large teacher network. These model compression methods can significantly reduce parameter size, computation cost and running time. More practical approaches and suggestions can be seen in Reference [40].

## 3. Architecture

The network architecture of our lightweight (LW) CNN consists of a LW bottleneck, classifier network, and segmentation decoder.

### 3.1. Depthwise Convolution

**Figure 1:**
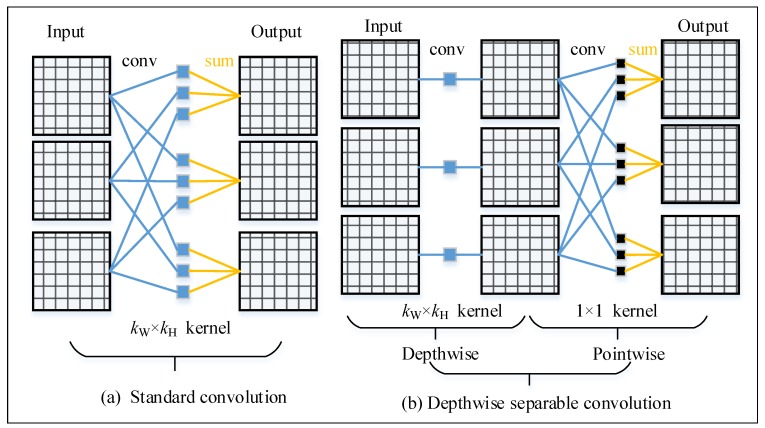
Standard convolution and depthwise convolution operations. A depthwise separable convolution is combined by a depthwise convolution and a 1×1 convolution (pointwise conv).

We call the regular convolution in deep learning as the standard convolution. Figure 1a shows the basic operations of standard convolution. In a standard convolution layer with $C_{in}$ input channels and $C_{out}$ output channels, each output feature map is the sum of the $C_{in}$ input feature maps convoluted by $C_{in}$ corresponding kernels. Many famous models such as Alexnet [41], VGG [42], and ResNet [20,43] use the standard convolution. The number of weights of standard convolution is:

(1) $$W_{\mathrm{std}}=C_{in}\times k_{W}\times k_{H}\times C_{out},$$

where $k_{W}\times k_{H}$ is the kernel size. To generate output feature maps with size $f_{W}\times f_{H}$, the computational cost is:

(2) $$MACs_{\mathrm{std}}=C_{in}\times k_{W}\times k_{H}\times C_{out}\times f_{W}\times f_{H},$$

where $k_{W}\times k_{H}$ is the kernel size and $f_{W}\times f_{H}$ is the output feature map size. MACs is Multiply-ACcumulate operations. The value of FLOPs evaluated by TensorFlow is roughly twice larger than of MACs.

Figure 1b displays the basic operations of depthwise convolution and depthwise separable convolution. Distinct from standard convolution, each output feature map of depthwise convolution is only the result of an input feature map convoluted by a single convolution kernel. The number of weights of a depthwise convolution is:

(3) $$W_{\mathrm{dw}}=k_{W}\times k_{H}\times C_{out}.$$

The computational cost of a depthwise convolution layer is:

(4) $$MACs_{\mathrm{dw}}=k_{W}\times k_{H}\times C_{out}\times f_{W}\times f_{H}.$$

Depthwise convolution is the key component of our backbone. It reduces the weights and computational cost by $C_{in}$ times. Depthwise separable convolution is the operation of depthwise convolution followed by a 1×1 convolution (pointwise convolution). In modern networks, depthwise separable convolution has been widely applied and achieved excellent performance, such as Xception [33] and MobileNets [23,25]. We also use the depthwise separable convolution in our decoder.

### 3.2. LW Bottleneck

**Figure 2:**
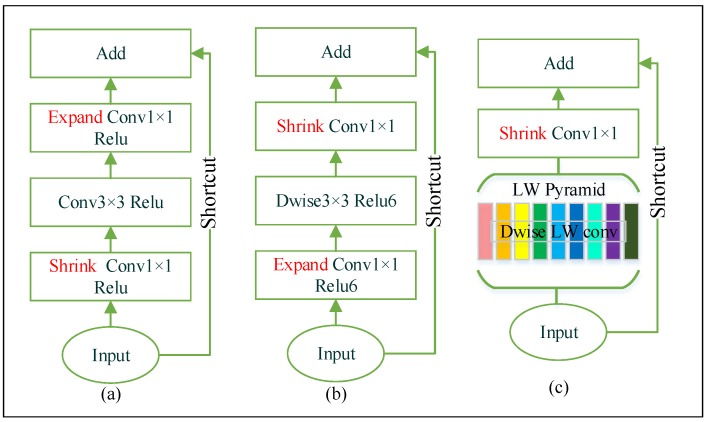
Our bottleneck structure (**c**) Vs ResNet (**a**) and MobileNetV2 (**b**). LW(LightWeight) Dwise(Depth-wise) convolution collecting rich features in difference size of receptive fields, building a LW pyramid. Different color in (**c**) means different convolution kernel size.

The deep bottleneck architecture of ResNet is introduced in Reference [20,43]. Figure 2a shows the structure of ResNet bottleneck. It substantially increases the feature map number, while decreasing the number of weights and the computational cost. Layer dimensionality impacts the manifold of interest in neural networks. In ResNet, the number of channels experiences a series of changes. Firstly, the network has high-dimensional inputs. Afterward, the number of channels significantly shrinks in the first pointwise convolution operation, then stays the same in the following standard convolution layer, and finally expands back to the input size in the next pointwise convolution. This structural design effectively reduces the computational cost of 3×3 standard convolution. Figure 2b shows the basic structure of MobileNetV2 invert residual bottleneck. On the contrary, the invert residual bottleneck has low-dimensional inputs, and the number of channels firstly expands then shrinks. The depthwise convolution is much lighter than standard convolution, making the thicker 3×3 convolution possible, and this structure proved to be more efficient in running memory and slightly increased the model accuracy.

Despite the depthwise convolution shrinking a remarkable number of weights, we attempt to reshape kernels for more performance improvement. In the LW bottleneck, the first layer has multiple parallel depthwise convolutions that will generate feature maps in different receptive fields, which is also equivalent to expanding channels to the next layer. Previous research has shown that the 5×1 and 5×1 convolutions can provide wider receptive fields and reduce weights with lager kernel techniques [44]. Based on the studies, some 3×3 kernels will be replaced with 1×k and k×1 to avoid redundant computation, and k will include 5. Specifically, the types of kernels are 5×1, 1×5, 3×1, 1×3, 2×1, 1×2, 2×2, and 3×3. Each kernel forms a depthwise convolution path, then the feature maps of the paths are concatenated. Afterward, a pointwise convolution is used to shrink the channels. The multiscale kernels build a pyramid with different receptive field sizes, which is structurally similar to the ASPP [31,45]. In a LW bottleneck, the number of weights is:

(5) $$\begin{array}{cc} W_{\mathrm{LW}}= & \left[5+5+3+3+2+2+2\times 2+3\times 3\right]\\ \; & \times C_{in}+8\times C_{in}\times 1\times 1\times C_{out}\\ = & \left(33+8\times C_{out}\right)\times C_{in} \end{array}$$

and a MobileNetV2 bottleneck with a expansion factor 6 is:

(6) $$\begin{array}{cc} W_{\mathrm{MB}}= & C_{in}\times 1\times 1\times 6\times C_{in}+6\times C_{in}\times 3\times 3\\ \; & +6\times C_{in}\times 1\times 1\times C_{out}\\ = & \left(54+6\times C_{in}+6\times C_{out}\right)\times C_{in}. \end{array}$$

In our network design, we hold the condition $C_{out}\leq 2\times C_{in}$, thus $W_{\mathrm{LW}}<W_{\mathrm{MB}}$.

Relu activation function is widely used for non-linear transformation and training acceleration [41]. Relu6 [46] is more robust when combining with low-precision computation. However, non-linearity will lead to significant information loss in the case of low-dimensional input [25]. Based on these studies, we only use Relu6 after the depthwise convolution but drop non-linear transformation after the pointwise convolution. We use stride rather than pooling in the depthwise convolution to enlarge the receptive field and reduce the feature dimension, since it is proved that fewer computational cost while no impact on the accuracy in using stride than pooling [47]. Residual shortcuts will be applied to prevent vanishing gradient once the input and output of the bottleneck have the same tensor shapes.

### 3.3. Backbone and Decoder

Table 2 describes our backbone implementation structure in detail. A block is the basic convolution unit, and it can either be a standard convolution or a bottleneck. In the table, *N* represents that the blocks are repeated by *N* times; *S* is the stride. *S* is used in the first depthwise convolution when the bottleneck blocks are stacked repetitively. Compared with MobileNetV2, our backbone is more compact in terms of the number of layers and blocks. For example, the MobileNetV2 has 53 convolution layers and 19 blocks, while our model has 21 and 12. Another improvement is on channel design, which leads to ∼1 M of weights reduction without hurting model performance. In our design, the output channels of the last layer are shrunk into 320, which is sufficient enough for surface defect inspection. We also use the width multiplier parameter for computation and accuracy tradeoff. Width multiplier is a tunable hyperparameter that is introduced by MobileNet and MobileNetV2 [25], when a multiplier is smaller than 1, the very last layer of the backbone will not be changed to maintain the accuracy performance. Width multiplier is used as a tunable hyperparameter for accuracy/performance tradeoffs, which can change network channels uniformly at each layer as MobileNets do.

**Figure 3:**
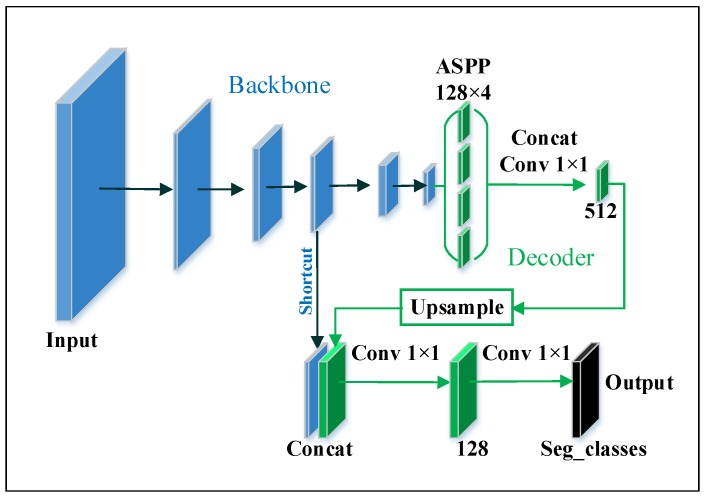
The proposed segmentation model. The decoder includes an atrous spatial pyramid pooling (ASPP), a bilinear upsampling, a series of concats and pointwise convolutions.

After the backbone, the operation procedure for classification and segmentation are different. In classification, the backbone is followed by a global average pooling layer, then a fully connected layer. For the segmentation task, a decoder is embedded, which is shown in Figure 3. The decoder consists of an Atrous Spatial Pyramid Pooling (ASPP) [31] and three pointwise convolution layers. In this decoder, the number of feature maps is fixed, and the output is the result of segmentation. The number of output channels equals the number of semantics, so we set channel number as 2—one for the foreground and another for the background. Specifically, the ASPP is composed of one pointwise convolution and three depthwise separable convolution layers. The kernels in depthwise separable convolution have the same size 3×3, but their atrous rates are different, which are 6, 12, and 18. The shortcut is from the 4th or the 5th block of the backbone, which corresponds to 1/4 and 1/8 of output size respectively.

### 3.4. Implementation Details

#### 3.4.1. Hyperparameter Settings

The network is implemented with TensorFlow. In both classification and segmentation tasks, we use softmax cross-entropy as the loss function and Adam optimizer as the adaptive gradient descent optimizer. The learning rate is 0.0001, which will exponentially decay 10% at every 10,000 steps. To speed up training, batch normalization layers are attached to each convolution layer, with decay = 0.9997 and epsilon = 0.00001. To avoid overfitting, we use L2 regularization in each convolution layer. The decay of L2 regularization is 0.0001.

#### 3.4.2. Gradual Training

Training deep neural networks on the surface defect datasets may have the issue of data imbalance—first, the image data are difficult to collect, because there only exists a small amount of the products with surface defect; second, the target features is difficult to detect, because the number of surface defect pixels are much less than background pixels. These characteristics can cause the background overfitting and the foreground underfitting. In the worst case, the neural network may predict the defective product as a “perfect product” because all the surface defect pixels may be “ignored” by the model. Besides, the size of different types of defects varies greatly, the gradient of tiny defects will be overwhelmed by the gradient of large defects or background gradient, causing the low accuracy of tiny defects.

We propose a gradual training strategy to address data imbalance. In the early stage of network training, we only train the images of defective products and zoom into the defect areas. The model is trained with constant input size, while the zoom ratio is linearly reduced to 1. Later, the images of perfect products are fed to the model. This strategy has proved to be a valid way to prevent the network from overfitting. It also improves model accuracy. Details of this training strategy will be expounded in Section 4.4.

## 4. Experiments

### 4.1. Datasets

#### 4.1.1. The Textures Dataset

We collect a dataset of surface texture (8674 images), which contains 64 classes from 3 public datasets. It can be accessed by https://github.com/abin24/Textures-Dataset. The images are uniformly resized into 331×331. Notably, 11 classes (3194 images) are from KTH surface datast (including KTH-TIPS and KTH-TIPS2) [48], 28 classes (4480 images) from Kyberge [49] dataset, and 25 classes (1000 images) from the UIUC dataset [50].

**Figure 4:**
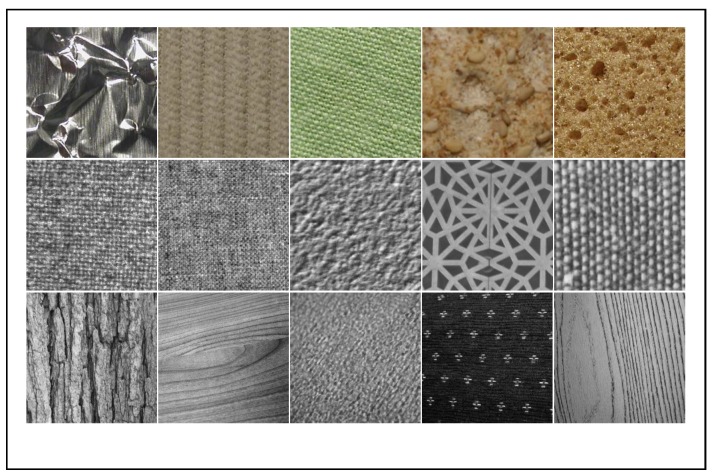
The textures dataset. Each image is a sample of one texture class, first row is from the KTH, second row from Kyberge, and third from UIUC.

Images in the Textures datasets include wood, blanket, cloth, leather, and so forth, these textures are very commonly seen in surface inspection problems. So, we believe that it can be used as a source dataset for network pretraining, and also can be used to evaluate the capacity of defect inspection models. Samples of this dataset are displayed in Figure 4.

**Figure 5:**
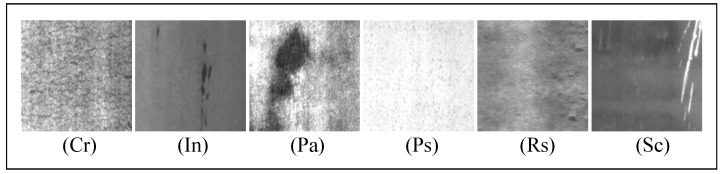
The 6 classes surface defects in NEU dataset.

NEU surface defect dataset [1] is a hot-rolled steel strip surface defect dataset including 6 classes (1800 images) of defects. Images are grayscale with uniform size 200×200. The steel defects include crazing (Cr), inclusion (In), patches (Pa), pitted surface (PS), rolled-in scale (RS), and scratches (SC), as shown in Figure 5. Images are captured in different exposure conditions, their intra-class defects exist large differences in appearance while the inter-class defects share some similar aspects, making the classification task challenging.

#### 4.1.2. The Dagm Dataset

**Figure 6:**
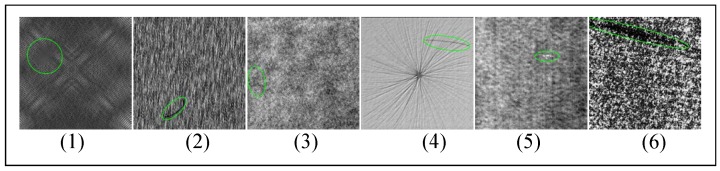
The DAGM dataset, the green ellipses are the provided coarse ground truths. (1)–(6) are the 6 classes of the surface in the dataset.

The DAGM 2007 dataset [51] has 6 classes of textured surfaces, it is evenly divided into training and test sets. As shown in Figure 6, defects are coarsely labeled with elliptical masks. In each class of the 6 surfaces, there are 575 images in the training set, about 80 with a defect and the rest are normal, and the test set shares this distribution.

**Figure 7:**
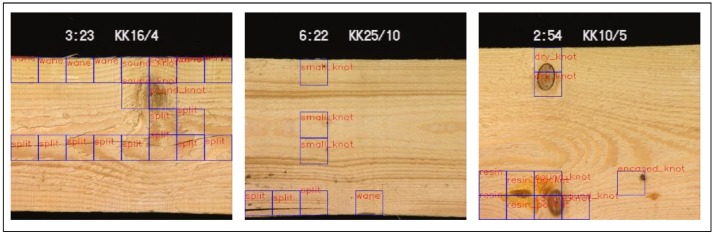
Samples in wood defect dataset, blue bounding boxes are the provided ground-truth, notice that the bounding box are not mapped well with the defects.

Wood defect dataset [5] is a surface defect dataset of dried lumber boards, containing 18 types of defects (839 images with size 488×512). Figure 7 shows defects and the corresponding ground truths with a set of bounding boxes. Some defects only appear in 2 or 3 images, such a severe imbalance of image distribution brings many difficulties to our work.

### 4.2. Classification on Textures

#### 4.2.1. Experiment Settings

We split the Textures dataset into two halves randomly, one for the training set, and another for the validation. We implement strong data augmentation to balance the image number of each class and avoid the effect of overfitting. In the training set, we generated 200 images for each class. Each augmented image is generated from a random combination of multiple data augmentation methods provided by imgaug library [52]. These data augmentation approaches include flipping, affine translation, crop, blur, Gaussian noise, illumination perturb, elastic transform, perspective transform, and piece-wise affine. The augmented images are mixed with the original images to build our final training set.

We compare our models with ResNetV2-50 [20], ShuffleNet [24], and MobileNetV2. The hyperparameter setting is the same as those described in Section 3.4. The input size is 331×331×3, and training for 90,000 steps with a batchsize of 64. Table 3 shows the experiment result, where @ represents Width multiplier which is mentioned at Section 3; *t* is the running time of a forward operation on an IPC that is equipped with an Intel i34010U CPU(1.7 GHz) and 4 GB memory; Acc is the test accuracy, defined as:

(7) $$Acc=\frac{C}{N}\times 100\%,$$

where *C* is the number of correct predictions in the test set and *N* is the number of all predictions in the test set.

#### 4.2.2. Impact of Parameter Size

A model with more weights is more likely to obtain higher accuracy. For example, MobileNetV2@1.4 performs better than MobileNetV2@0.35; ours@0.75 performs better than ours@0.35. However, ours@0.75 obtains higher accuracy than MobileNetV2@1.4 with fewer weights when the models are trained from scratch (random initialization).

In the experiments, the fine-tuned MobileNetV2@1.4 obtains the highest accuracy. Regardless of the good accuracy performance of the MobileNetV2@1.4 and ResNetV2-50, these models contain a large number of parameters (and operations) which require GPUs. However, an ideal ASI model should not only perform well inaccuracy but also takes less computational resources, because many ASI platforms usually are not equipped with such expensive hardware devices. After exploring the trade-off between accuracy and efficiency, we give up on embedding these large-parameter models.

#### 4.2.3. Impact of Fine-Tune

All the backbone networks in this paper are either trained from scratch (random initialization) or fine-tuned from pretained parameters. As shown in Table 3, we pretrain our model on Oxford Flower102 [53] and ImageNet. We obtain higher accuracy improvement using fine-tune strategy than simply stacking more weights or layers. Compared with the random initialized ResNetV2-50 and MobileNetV2@1.4, ours@0.75 model (ImageNet pretrained) has much fewer weights and FLOPs, but higher accuracy. The fine-tune strategy can enhance accuracy by 1∼2 %.

**Figure 8:**
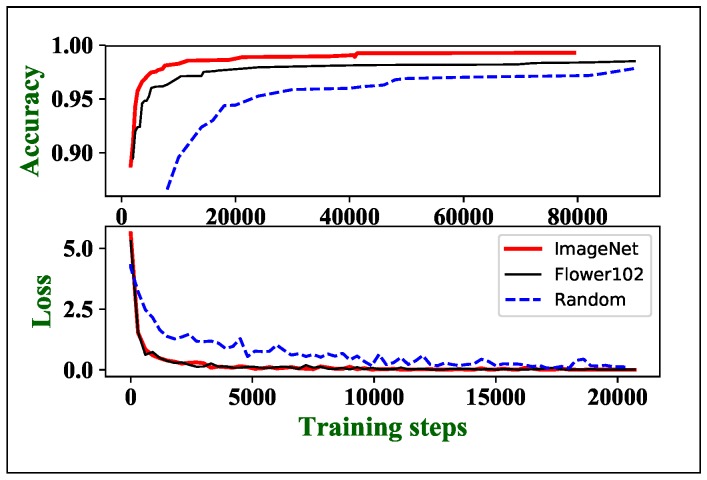
The accuracy and loss of different parameters initialization methods (our@0.35).

Fine-tune can also accelerate convergence. As shown in Figure 8, the Cross-Entropy losses of pretrained models decrease much faster than the random initialization ones. The ImageNet pretrained MobileNetV2@1.4 obtains the highest validation accuracy (99.93%) with only 4000 training steps, which is 22.5 times faster than the random initialization. Our experiment also shows that transferring the features of the larger scale source dataset is more generalization. As shown in Figure 8, ImageNet pretrained model has higher validation accuracy than Flower102 pretrained model at the same training loss. The ImageNet pretrained model has also higher accuracy improvement as seen in Table 3. However, it takes days to weeks to pretrain a designed model on ImageNet with a high-performance computer equipped with exceptionally strong GPUs and over 130 GB disk storage. Since the accuracy improvement of Flower102 pretraining is competitive to ImageNet pretraining, a much smaller source dataset can be used as a compromise when computing resources are limited.

Our model (ours@0.75) is ∼4.9 times faster than MobileNetV2@1.4, with only ∼1/10 weights, ∼1/4 FLOPs and a slight (∼0.41%) accuracy decrease. Compared to the models with similar FLOPs size, our models also have competitive accuracy to ShuffleNet@0.25 and MobileNet@0.35, but much faster speed on low power CPU.

### 4.3. Classification on NEU

Each class of NEU dataset has 300 200×200 images, they are randomly split into 150 training images and 150 test images. We use accuracy defined in Equation (7) as evaluation metric, and the classification result can be seen in Table 4.

SCN + SVM [1] is the first model using the NEU dataset. In this model, a translation and scale invariant wavelet method called scattering convolution network (SCN) is used to extract texture features; support vector machine (SVM) is used as the classifier. Their experiments proved that SCN performs better than other methods on extracting features, and SVM performs better than the Nearest Neighbor Classifier on this dataset. This method achieves 98.60% accuracy. Reference [2] introduces an end-to-end CNN for this surface defect classification task. Their work proves that data augmentation on this dataset is crucial for performance improvement, their model gets a better accuracy at 99.05%. Ruoxu, Ren et al. [54] used an ImageNet pretrained CNN named Decaf [55] as a fixed peculiarity extractor, they remove the fc7, fc8 layer, freeze the layers before fc6, then trained a multinomial logistic regression (MLR) classifier. This transfer learning method helps them reach a higher accuracy at 99.27%. Larger-size models can fit the dataset better, mentioned in Reference [56], Wide Residual Networks (WRN) [57] are used, and the WRN-28-20 gets the best-reported result at 99.89%. However, as shown in the Table 4, WRN has so many weights to train that results in a massive computation cost during training, as long as 2 days as their report, even if the input images were resized to 32×32.

We apply strong data augmentation to generate sufficient data for network training. Each class contains 9000 images after data augmentation, all the augmentation methods mentioned in Section 4.2.1 is used in this case. We train ShuffleNet@0.25, MobileNetV2@0.35 and ours@0.35 at this augmented dataset with 200×200 input size and a batch size of 64, losses and hyperparameters are the same as those described in Section 3.4.

Both MobileNetV2@0.35 and the ours@0.35 achieve exceptionally high performance with the best record of 100% test accuracy. However, compared to the minor improvement on accuracy, it is more worth pointing out that the pretrained models accelerate convergence. For instance, the ImageNet pretrained MobileNetV2@0.35 and ours@0.35 takes only 1.2 K–1.6 K training steps to converge (99% + test accuracy). In the setting of random initialization, the training time of ShuffleNet@0.25, MobileNetV2@0.35 and the ours@0.35 is almost identical, which takes around 4 h (60K steps) on a GTX 1080 GPU, much faster than the 48 h of WRN-28-10. Our model has the least parameter, computational cost and running time but the highest accuracy in the NEU dataset.

### 4.4. Segmentation on DAGM

#### 4.4.1. Comparison Models

We use the mAcc (mean Accuracy) in Reference [14] as our evaluation metric, which is defined as:

(8) $$mAcc=\frac{TPR+TNR}{2}$$

and TPR (True Positive Rate), TNR (True Negative Rate) are defined as:

(9) $$TPR=\frac{TP}{TP+FN},\;TNR=\frac{TN}{TN+FP}.$$

mAcc is an image-level evaluation metric, without considering pixel-level segmentation accuracy. Specially, the mAcc in Table 5 is the mean mAcc of all the 6 type textures of the test set.

In Reference [13], 32×32 image patches and their 64×64 context patches are randomly extracted from the training images, and fed to a context-aware CNN(CA-CNN) classifier together. All test images are classified into 12 classes(6 background textures and 6 defects) with the 32×32 patches. Context information is critical to increasing the mAcc this sliding window classifier. In Reference [14], the problem of segmentation is also treated the defect segmentation as a classification task in 32×32 patches, with CNNs and sliding windows (CNN + SW). There also exist some attempts to select an optimal CNN structure among different depths and widths between operation time and accuracy trade-off. In Reference [58], the segmentation strategy includes two stages: first, an FCN is used to segment defects; second, the 32×32 or 64×64 patches are cropped around the defect detected by the first stage and a CNN is used as a foreground-background classifier to reduce false alarms. These three approaches use very small patches. However, the elliptical ground contains tremendous non-defective areas (as shown in Figure 6(2) and (4)). To extract positive samples, more accurate manual re-label ground truths are necessary. The ever-changing texture background makes a universal set of background patches to train the network, in Reference [14] 324,000 training patches are cropped to ensure model accuracy. So these sliding window based models are unstable and hard to train.

In Reference [15] a input image is cropped to 9 224×224 sub-images, and an ImageNet-pretrained VGG-16 [42] is fine-tuned. The whole image is classified by the vote result of 9 sub-images. Rich features learned from ImageNet push the VGG-16 well-fit to the DAGM and get the best mAcc(99.93%) overall. However, the parameters of a VGG-16 are 134.31 M, which causes very large computational cost—9024 M FLOPs in one sub-image, 9 times FLOPs are needed in an entire input image.

In Reference [29], an end-to-end segmentation-classification multi-task compact CNN(C-CNN) is used. Different from the ordinary segmentation model, they use a mean squared error (MSE) loss to treat the defect segmentation as a regression problem and use heat maps as segmentation results. The training process has two stages: first, only the regression FCN is trained; second, the layers related to FCN are frozen and the classifier is trained only. This training strategy enables the model to fit a good classifier without affecting the segmentation accuracy. The mAcc of this model is 99.43% with small computational cost and very few parameters.

#### 4.4.2. Proposed Model

The DAGM dataset contains 6 classes of defects. Noticeably, the full input image size is 512×512, but the width of the smallest defects only consists of 3–4 pixels. We treat this defect inspection as a binary image segmentation task and the elliptical mask as the supervise ground truth. We build a segmentation neural network with the encoder-decoder illustrated in Figure 3, in which input size = 512×512, output stride = 8, and output size = 64×64. The encoder backbone is our LW backbone, ShuffleNet or the MobileNetV2, and the decoder shortcut is the 5th layer output of the backbone. The training batch size is set to 12, losses and hyperparameters are the same as those described in Section 3.4. The outputs of these semantic segmentation model are two 64×64 binary images, one for the foreground (defect) and another for the background. When we evaluate the mAcc in the test set, if any white pixel appears in the predicted foreground, the image will be treated as a positive sample, otherwise, it will be predicted as normal (negative). The training set is augmented by methods described in Section 4.2.1, since some spatial augmentation (affine/flip/crop/etc.) will change the locations of the defects, the same spatial augmentation transform will be applied to the ground truth masks as well. Each type of these texture surface will be augmented to 6000 images.

**Figure 9:**
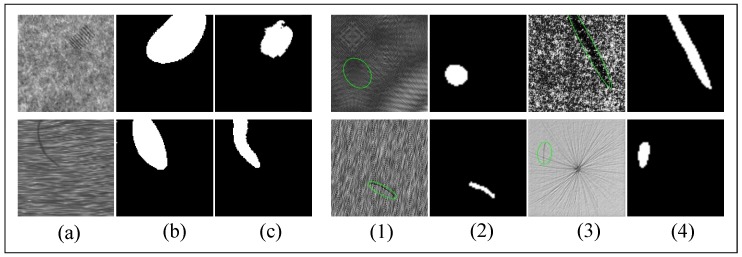
Results of DAGM dataset. (**a**): At the early training steps, training images are zoomed to the defect, (**b**) are the label of (**a**), (**c**) are the predicted images of the model. (1), (3) are the full-scale image, (2), (4) are the prediction of the well-trained model, predict masks are more accurate than the ground truths sometimes.

The proportion of training images with defects and non-defects is 1/7, and some tiny defects only consist of 3-4 pixels width while the image size is 512×512. Such severe imbalance is highly likely to cause low accuracy of tiny defects because of foreground under-fitting. We use the gradual training strategy mentioned above (Section 3.4), to address the issue of the severe imbalance distribution of DAGM. First, only the images with defects are trained at the beginning, and these images are zoomed to the defect area. Figure 9a presents an example of the process. The zoom ratio is linearly reduced from 3.6 to 1 at the first 20 K steps, then the model continues to train with the original input size. The images without defects are fed to the model after 80 K steps. These setups are empirically designed, and our experiments prove that this training strategy can help obtain better performance on surface defect inspection.

As shown in Table 6, gradual training improves $TPR_{2}$ of the tiny defect (the True Positive Rate of the second type texture surface), but cannot improve the mAcc. This conservative training strategy enables the network to learn the feature of tiny defects, to avoid foreground underfitting and result in predict all images as the background. Together with ImageNet pretraining, 4.77% $TPR_{2}$ and 0.61% mAcc improvement can be achieved. Pretraining is the key process accuracy improvement, pretraining on small datasets like Flower102 or Textures is competitive to large scale dataset (ImageNet).

Table 5 shows the statistic of model performance on DAGM dataset. Ours@0.35 model costs the least computation with the least weights, while still achieves a mAcc of 99.43%. ImageNet pretrained MobileNetV2@0.35 with our LW decoder get a very high mAcc (99.90%). In contrast to the FCN+CNN [58], CA-CNN [13], CNN [14] and other patch-based models, our model does not require finer ground truth. Universe set of background patches is also no longer needed, and the end-to-end prediction does not require any post-processing steps. As shown in Figure 9, our model makes a fine prediction of elliptical defect masks, and sometimes the outputs are more accurate than the ground truths. This phenomenon indicates that the model has learned the feature of defects rather than overfitting the training set.

Ours@0.35 is 3 times faster than the MobileNetV2@0.35 on a low-cost IPC, which make ∼5 fps surface defect inspection is possible, with only 0.47% mAcc drop. Ours@0.35 has same mAcc with the ShuffleNet@0.25 but 1.7 times faster on the IPC. When using a GPU, ours@0.35, ours@0.75, ShuffleNet@0.25+ours decoder and MobileNetV2@0.35+ours decoder can inference a 512×512 image within 8 ms.

### 4.5. Segmentation on Wood Defects

**Figure 10:**
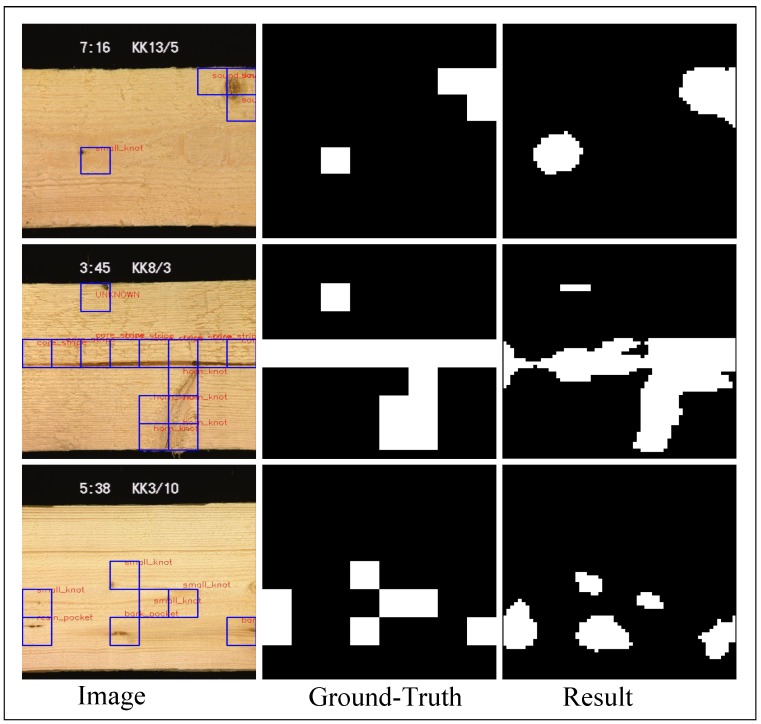
Segmentation results of Wood defect dataset.

Each image in the Wood defect dataset has a set of bounding boxes of the same size, and a label indicating the defect type inside these bounding boxes (shown in Figure 7 and Figure 10). These evenly distributed bounding boxes sometimes do not match the defects well. We use ours@0.75 as the backbone, which also pretrained from DAGM dataset, as the encoder. The output stride is 8, the input size is 488×512×3, and the defect bounding boxes as the ground truth. Half of the images are used as the training set and the rest as the test set, each of the 18 type defect is augmented to at least 6000 images with the same method used in DAGM dataset, to reduce the effects of data imbalance. We also adopt all the hyperparameters and gradual training strategy the same as we used in the DAGM dataset.

We adopt the Accuracy and Error escape rate defined in Reference [54], the Accuracy is defined as:

(10) $$A=1-\frac{\left(N_{\mathrm{lab}}-N_{\det\_\mathrm{lab}}\right)+\left(N_{\det}-N_{\det\_\mathrm{lab}}\right)}{N}$$

And Error escape rate is defined as:

(11) $$E=1-\frac{N_{\det\_\mathrm{lab}}}{N_{\mathrm{lab}}}$$

where the $N_{\mathrm{lab}}$ is the number of defect patches (bounding boxes), $N_{\det}$ is the number of detected defect patches, $N_{\det\_\mathrm{lab}}$ is the correctly detected defect numbers, and *N* is the total number of patches. High accuracy (*A*) and low Error escape rate (*E*) is expected.

In Reference [54], an ImageNet pretrained Decaf is used as the fixed feature extractor, and a multinomial logistic regression (MLR) classifier is trained with patches. Heatmaps are generated from the predict probabilities of the MLR, and Otsu’s method together with Felzenswalb’s segmentation is used to generate binary segmentation results. They found that patch size and patch stride will be a greatly affect the result, small patches will not include enough information, and small stride will lead to high computational cost, while large patch size or stride will lead to imprecise segmentation result. And in this dataset, they choose a patch size roughly the same as the size of the defect (50×50) and a stride of 10. They got the best-reported result in all the defect types, which has the highest *A*, and the lowest *E*.

**Figure 11:**
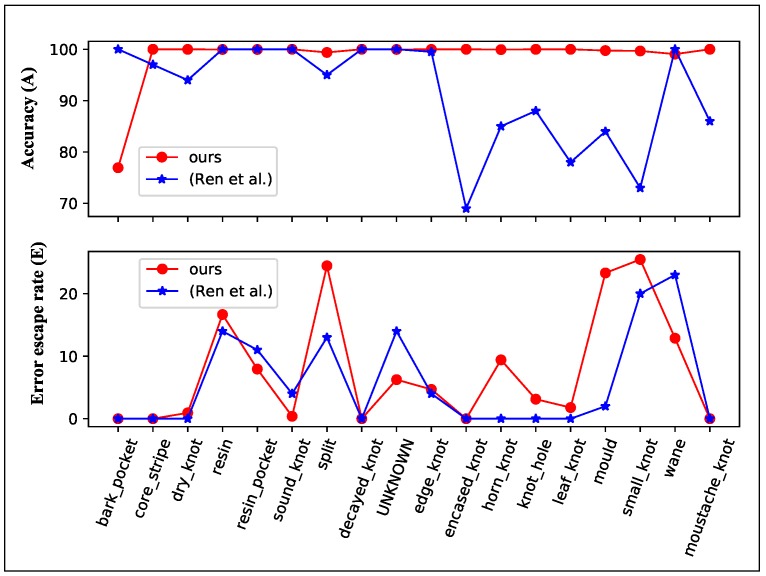
Accuracy and Error escape rate of Wood defect dataset.

We also treat this task as a binary segmentation, the segmentation result can be seen in Figure 10, our model fits the defect better than the provided bounding boxes. The *A* and *E* of our model and the best-reported result of Reference [54] is shown in Figure 11. Our model has much higher accuracy than the Decaf and similar Error escape rate. Our model is much faster than the reported segmentation time in Reference [54], they use a 24 core workstation and 2 min to generate heatmaps per image, our model tests an image in 397 ms with an Intel i3-4010U(1.7 GHz) CPU or 27 ms with a GTX1080 GPU. Our model has no patch size selection process and no post-process, differ from the segmentation method in Reference [54].

## 5. Conclusions

In this paper, we introduce a novel compact CNN model with low computation and memory cost as well as explore the availability that if one model can be applied on cross-product surface defect inspection. We modify classic backbone architecture into the LW backbone via switching channel expansion order and customizing small kernels. Meanwhile, we observe that some strategies, e.g., transfer learning and zoom-in training, are critical to building the networks of tiny defect inspection. By evaluating the tradeoff between accuracy and computation cost, our model outperforms other advanced models with the least computation on four defect datasets. In contrast to the traditional approaches, our model gets rid of hand-crafted features and patch size selection. Compared with modern CNNs, the LW model has less than 1 M weights, which is no longer needed to run on expensive GPUs.

We record the experiment results on an IPC equipped with an Intel i3-4010U CPU (1.7 GHz). The inference time of a 200×200 image in the NEU dataset is ∼29 ms, a 331×331 image in the Textures dataset is ∼140 ms (@0.75, 99.33% Acc) and a 512×512 image in DAGM dataset takes 217 ms (@0.35, 99.43% mAcc). The inference time will be <30 ms if using a GPU. This lightweight, end-to-end compact model is promising to be used in low power consumption real-time surface inspection systems or smartphone apps.

## Figures and Tables

**Table 1:** The top-1 accuracy of the models on the ImagNet. *F* refer to FLoating-point OPerations (FLOPs) and *W* to Weights. **bold** means the best performance.

Model	Layers	*F* (M)	*W* (M)	Top-1 Acc (%)
AlexNet	8	1386	49.43	57.2
VGG-16	16	9040	138.35	71.5
ResNetV2	50	7105	25.57	75.6
ResNetV2	101	14,631	44.58	77.0
ResNetV2	200	29,165	64.73	79.9
Inception-V4	-	12,479	42.65	**80.2**
MobileNetV2@1.4	53	**1189**	**6.11**	75.0

**Table 2:** The backbone of light weight (LW) network with a $331^{2}\times 3$ input.

Input	Operator	$C_{\mathrm{out}}$	*S*	*N*	Block
$331^{2}\times 3$	3×3 Conv2d	32	2	1	1
$166^{2}\times 32$	1×1 Conv2d	16	1	1	2
$166^{2}\times 16$	LW bottleneck	24	2	2	3–4
$83^{2}\times 24$	LW bottleneck	32	2	2	5–6
$42^{2}\times 32$	LW bottleneck	32	2	1	7
$21^{2}\times 32$	LW bottleneck	64	2	4	8–11
$11^{2}\times 64$	1×1 Conv2d	320	1	1	12

**Table 3:** Model performance on the Textures dataset. *F* refer to FLOPs, *W* to Weights, *t* to running-time, and Acc to Accuracy.

Model	Pretrain	*F* (M)	*W* (M)	*t* (ms)	Acc (%)
ShuffleNet@0.25	-	196	0.19	254	97.02
MobileNetV2@0.35	-	267	0.48	259	97.72
MobileNetV2@1.4	-	2684	4.43	695	97.56
ResNetV2-50	-	16082	23.65	893	**99.03**
ours@0.35	-	**76**	**0.06**	**75**	97.51
ours@0.75	-	246	0.14	140	98.71
ShuffleNet@0.25	ImageNet	196	0.19	254	99.33
MobileNetV2@0.35	ImageNet	267	0.48	259	99.63
MobileNetV2@1.4	ImageNet	2684	4.43	695	**99.93**
ResNetV2-50	ImageNet	16082	23.65	893	99.60
ours@0.35	ImageNet	**76**	**0.06**	**75**	99.29
ours@0.75	ImageNet	246	0.14	140	99.52
ShuffleNet@0.25	Flower102	196	0.19	254	99.27
MobileNetV2@0.35	Flower102	267	0.48	259	99.50
MobileNetV2@1.4	Flower102	2684	4.43	695	**99.73**
ResNetV2-50	Flower102	16082	23.65	893	99.58
ours@0.35	Flower102	**76**	**0.06**	**75**	98.48
ours@0.75	Flower102	246	0.14	140	99.33

**Table 4:** The accuracy of the NEU dataset.

Model	Pretrain	*F* (M)	*W* (M)	*t* (ms)	Acc (%)
SCN + SVM	-	-	-	-	98.60
CNN(Yi Li)	-	**400**	**0.46**	-	99.05
Decaf + MLR	ImageNet	1078	28.58	-	99.27
WRN	-	115557	144.8	-	**99.89**
ShuffleNet@0.25	-	52	0.19	126	99.89
MobileNetV2@0.35	-	102	0.40	116	99.89
ours@0.35	-	**28**	**0.04**	**29**	**100**
ShuffleNet@0.25	ImageNet	52	0.19	126	99.89
MobileNetV2@0.35	ImageNet	102	0.40	116	**100**
ours@0.35	ImageNet	**28**	**0.04**	**29**	**100**

**Table 5:** The mean accuracy of the DAGM dataset.

Model	Pretrain	*F* (M)	*W* (M)	*t* (ms)	Acc (%)
FCN + CNN	-	-	-	-	95.99
CA-CNN	-	-	-	-	96.44
CNN + SW	-	-	-	-	99.20
C-CNN	-	**2280**	**1.27**	-	99.43
VGG	ImageNet	9024	134.31	-	**99.93**
ShuffleNet@0.25 + ours decoder	-	1126	0.64	371	98.99
MobileNetV2@0.35 + ours decoder	-	1630	1.40	591	98.99
ours@0.35	-	**923**	**0.55**	**217**	98.86
ours@0.75	-	1312	0.60	369	**99.46**
ShuffleNet@0.25 + ours decoder	ImageNet	1126	0.64	371	99.40
MobileNetV2@0.35 + ours decoder	ImageNet	1630	1.40	591	**99.90**
ours@0.35	Textures	**923**	**0.55**	**217**	99.40
ours@0.35	ImageNet	**923**	**0.55**	**217**	99.43
ours@0.75	Textures	1312	0.60	369	**99.79**
ours@0.75	ImageNet	1312	0.60	369	99.67

**Table 6:** Impact of the gradual training and pretraining on tiny defect.

Model	Gradual Training	Pretrain	${\mathrm{TPR}}_{2}\;\left(\%\right)$	mAcc(%)
ours@0.35			90.47	98.82
ours@0.35	✓		94.05	98.86
ours@0.35	✓	✓	**95.24**	**99.43**
